# Drought and salt stress mitigation in crop plants using stress-tolerant auxin-producing endophytic bacteria: a futuristic approach towards sustainable agriculture

**DOI:** 10.3389/fpls.2024.1422504

**Published:** 2024-07-02

**Authors:** Sadananda Mal, Shweta Panchal

**Affiliations:** School of Biosciences and Technology, Vellore Institute of Technology, Vellore, India

**Keywords:** climate change, zero hunger, plant microbiome, phytohormones, IAA, bioinoculants, abiotic stress

## Abstract

Abiotic stresses, especially drought stress and salt stress in crop plants are accelerating due to climate change. The combined impact of drought and salt is anticipated to lead to the loss of up to 50% of arable land globally, resulting in diminished growth and substantial yield losses threatening food security. Addressing the challenges, agriculture through sustainable practices emerges as a potential solution to achieve Zero Hunger, one of the sustainable development goals set by the IUCN. Plants deploy a myriad of mechanisms to effectively address drought and salt stress with phytohormones playing pivotal roles as crucial signaling molecules for stress tolerance. The phytohormone auxin, particularly indole acetic acid (IAA) emerges as a paramount regulator integral to numerous aspects of plant growth and development. During both drought and salt stress conditions, auxin plays crucial roles for tolerance, but stress-induced processes lead to decreased levels of endogenous free auxin in the plant, leading to an urgent need for auxin production. With an aim to augment this auxin deficiency, several researchers have extensively investigated auxin production, particularly IAA by plant-associated microorganisms, including endophytic bacteria. These endophytic bacteria have been introduced into various crop plants subjected to drought or salt stress and potential isolates promoting plant growth have been identified. However, post-identification, essential studies on translational research to advance these potential isolates from the laboratory to the field are lacking. This review aims to offer an overview of stress tolerant auxin-producing endophytic bacterial isolates while identifying research gaps that need to be fulfilled to utilize this knowledge for the formulation of crop-specific and stress-specific endophyte bioinoculants for the plant to cope with auxin imbalance occurring during these stress conditions.

## 1 Introduction

In the last few decades, the evidence of climate change due to harsh human activities has threatened global biodiversity, especially of plants because of their sessile nature (Penuelas et al., 2002; Rosbakh et al., 2017; Anthelme et al., 2021; Vecerova et al., 2022). Plants depend only on internal mechanisms to withstand stress and modifications in their external surroundings (Esmon et al., 2005; Knudsen et al., 2018). Plants encounter two primary forms of stress: biotic, caused by various pathogenic bacteria, fungi, nematodes, oomycetes, and herbivores, and abiotic, arising from factors like salinity, drought, radiation, heavy metals, and extreme temperatures (Gull et al., 2019). Among these, drought, and salt stress have affected almost 2000 million hectares of land globally (Beltagy and Madkour, 2012). Drought alone has an impact on 45% of the global agricultural land, and 19.5% of irrigated agricultural areas are classified as saline (Abdelraheem et al., 2019). Consequently, crop production is hindered on a global scale, posing a threat to global food security (Fahad et al., 2017). According to the Food and Agriculture Organization (FAO), over 870 million people worldwide are affected by food insecurity, hindering progress towards achieving “Zero Hunger”, one of the 17 Sustainable Development Goals outlined by the International Union for Conservation of Nature (IUCN) to be achieved by 2030 (FAO et al., 2022).

During drought and salt stress, plants experience water scarcity, ion toxicity, phytohormone imbalances, and increased production of reactive oxygen species (ROS), leading to considerable decreases in crop growth rate and the accumulation of biomass (Das and Roychoudhury, 2014). Plants deploy a myriad of mechanisms, encompassing osmotic adjustment, antioxidant defense, stomatal regulation, root system modification, transcriptional regulation, and phytohormone regulation, to effectively address stress. Phytohormones play pivotal roles serving as crucial signaling molecules for stress tolerance by activating multiple signaling pathways. Auxin, gibberellin (GA), cytokinin, ethylene, jasmonic acid (JA), and salicylic acid (SA) constitute the primary phytohormones crucial for regulating diverse biochemical and physiological processes governing plant growth and stress response (Abobatta, 2020; Sabagh et al., 2022). Auxin plays crucial roles during stress like improving root architecture by increasing lateral root number, expression of stress-related genes, metabolic homeostasis, and ROS detoxification (Shi et al., 2014). However, during both drought and salt stress, plants exhibit diminished auxin levels and reduced expression of auxin transporters which results in a disruption of auxin transport and distribution, leading to lowered stress tolerance (Park et al., 2007; Sun et al., 2008; Du et al., 2012; Liu et al., 2015). Crops can acquire supplementary auxin through various alternative methods. While the application of synthetic auxins is a prevalent practice, it comes with several drawbacks. These compounds exhibit high toxicity and are irritating to the eyes, skin, and respiratory system of farmers. Furthermore, their use can lead to unregulated or irregular plant growth tendencies, such as epinasty (Bhojwani, 2012; Keswani et al., 2020) Another alternative approach involves the contribution of plant-associated beneficial microorganisms, which have been reported to augment auxin levels in plants (Arshad and Frankenberger, 1991; Nassar et al., 2005; Tsavkelova et al., 2007; Shi et al., 2009; Keswani et al., 2020; Iqbal et al., 2023). Endophytic bacteria have been documented to promote plant growth in various crops including rice (Walitang et al., 2017), wheat (Yandigeri et al., 2012; Patel and Archana, 2017), maize (Riggs et al., 2001), potato (Nowak et al., 1995; Pavlo et al., 2011), cucumber (El-Tarabily et al., 2009; Shaalan et al., 2021), cotton (Bashan et al., 1989; Mohamad et al., 2022; Verma et al., 2022), tomato (Pillay and Nowak, 1997; Agarwal et al., 2020).

Endophytic bacterial diversity has been documented across numerous plant species with the Proteobacteria phylum being the most diverse and predominant (Santoyo et al., 2016; Afzal et al., 2019). The bacterial genera most frequently isolated include *Bacillus, Microbacterium, Pantoea, Burkholderia, Micrococcus and Stenotrophomonas*, with *Pseudomonas* and *Bacillus* being the prominent ones (Hallmann et al., 1997; Chaturvedi et al., 2016; Afzal et al., 2019).

**Figure 1:**
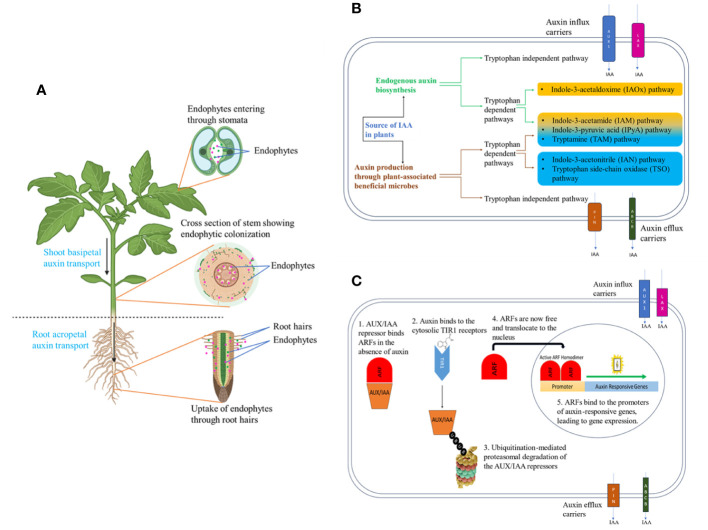
Endophytes of plants and auxin biosynthesis and signaling in plants. **(A)** The direction of polar auxin transport in plants and colonization of auxin-producing endophytes within the plant are depicted, **(B)** The biosynthesis pathways for both endogenous plant auxin and auxin produced by beneficial microbial associations, as well as the carriers responsible for auxin influx and efflux, are illustrated **(C)** Auxin signaling cascade in plants: 1. when auxin is absent, AUX/IAA repressors bind Auxin Response Factors (ARFs) in the cytosol, 2. When auxin is present, AUX/IAA repressors are degraded and ARFs move to the nucleus to activate auxin-responsive genes.

Endophytes have been isolated from various tissues of the plant, with roots harboring the maximum number owing to their proximity to a microbe-rich soil environment (**Figure 1A**). Root rhizodermis cells produce a variety of metabolites, including sugars, purines, amino acids, inorganic ions, and vitamins while root cap cells produce polysaccharide mucilage, facilitating their selective entry into the plant interior (Quadt-Hallmann et al., 1997; Dakora and Phillips, 2002; Bulgarelli et al., 2013; Frank et al., 2017). Endophytes gain access to aerial tissues such as flowers, fruit, stems, and leaves through natural openings like stomata as well as via accidental wounds (Frank et al., 2017; Synek et al., 2021). Endophytes can be vertically transferred through seeds and pollen to the next generation and horizontally transferred by colonizing root and aerial tissues. Recent literature establishes the role of the plant microbiome, especially endophytic bacteria in boosting plant growth, and one of the mechanisms is by elevating auxin levels within plants in response to stress (Kushwaha et al., 2020; Siddique et al., 2022; Kaur and Karnwal, 2023).

This review aims to provide a comprehensive outlook on involvement of auxin in drought and salinity stress, focusing on the disruptions in auxin biosynthesis, transport, and signaling under these conditions. To address these imbalances, potential stress-tolerant endophytic bacteria capable of producing auxin are highlighted. However, translation of this knowledge is currently lacking due to certain limitations. Efforts to create crop-specific and stress-specific bioformulations are minimal. In this review, we try to outline a roadmap to drive these results into potentially useful products. We will discuss the efficient use of these bacterial isolates in the formulation of bioinoculants and how technological advancements in research can further enhance this approach towards sustainable agriculture.

## 2 Methodology

For this review, articles were sourced from the electronic databases Scopus, Web of Science, and Google Scholar. The search encompassed the entire span of these databases’ archives up to February 2024, followed by a comprehensive screening that involved manually reading the title and abstract of the retrieved literature.

The search for relevant articles was conducted using the following keywords: plant stress, auxin, drought stress, salt stress, endophytic bacteria, stress tolerance, plant growth promotion, bioinoculants, sustainable agriculture, nanotechnology, and nanoparticles. These terms were strategically combined using the Boolean operators “AND” and “OR” to refine the search scope to the topic of interest.

Only studies that evaluate auxin production and plant growth promotion capabilities of endophytic bacteria under conditions of drought and salt stress are included. Research focused on stress mitigation strategies of endophytic bacteria, rather than auxin production, and studies published in non-indexed journals are excluded.

## 3 Auxin in plants

Indole-3-acetic acid (IAA), Indole-3-butyric acid (IBA), 4-chloroindole-3-acetic acid (4-Cl-IAA), and phenylacetic acid (PAA) are produced within plants, making them exclusive auxins categorized as “endogenous auxins” (Went and Thimann, 1937; Koepfli et al., 1938; Porter and Thimann, 1965). Furthermore, synthetic auxins remain pivotal as herbicides, with compounds such as 2,4- dichlorophenoxyacetic acid being widely utilized worldwide. The regulation of growth and development mediated by auxin involves multiple processes, including its biosynthesis, transport, perception, signaling, and conjugation, all working in concert to coordinate the plant’s response. The process of auxin biosynthesis in plants is intricate and involves multiple pathways, as depicted in **Figure 1B**. According to their physiological status, different plants use different pathways but there are shared fundamental mechanisms across plant species due to the critical role of auxin in the plant life cycle (Mano and Nemoto, 2012). Auxin transportation across plant cells involves a combination of membrane diffusion and carrier-mediated transport mechanisms. **Figure 1B** highlights some of the influx and efflux carrier proteins involved in this process (Kramer and Bennett, 2006). Auxin can move both basipetally and acropetally from one part of the plant to another with the assistance of these carrier proteins (**Figure 1B**) (Blakeslee et al., 2005). Auxin is perceived by a cytosolic receptor known as TIR1, initiating a complex signaling cascade as depicted in **Figure 1C** leading to the regulation of auxin-responsive genes.

Among the natural auxins, IAA stands out as the primary auxin in plants, playing a critical role in regulating many facets of plant growth and development. IAA is involved in root development initiating lateral root and adventitious root formation (Yu et al., 2020), cell elongation (Cleland, 1987), gametophyte development (Zhang and O’Neill, 1993), development of fruit (Pattison et al., 2014), and tropisms (Muday, 2001). Endogenous auxin in plants exists in both active and inactive forms, with the active forms playing a crucial role in signaling and constituting the pool of endogenous free auxin. For instance, only approximately 25% of the total quantity of IAA is present in its active form, while the majority exists as inactive forms like ester and amide conjugates, which do not actively participate in signaling (Ludwig-Müller, 2011). During abiotic stresses, the formation of these conjugates increases, leading to a decrease in the quantity of endogenous free auxin. To cope with this reduction, plant-associated endophytes supply free auxin during stress conditions, aiding plants in maintaining adequate auxin levels. Several auxin biosynthesis pathways have been identified in these plant growth-promoting endophytes (**Figure 1B**, Sukumar et al., 2013; Jahn et al., 2021).

### 3.1 Auxin and drought stress

When plants are subjected to drought stress, it typically leads to a notable decrease in the growth and yield of various crops. Auxin plays critical roles in mitigating drought stress through various mechanisms. In *Arabidopsis*, auxin upregulates antioxidant enzymes including superoxide dismutase (SOD), peroxidase (POD), catalase (CAT), and glutathione reductase (GR), and helps in decreasing the reactive oxygen species generated due to the stressful conditions. Auxin also upregulates different abiotic stress-related gene expressions like *RAB18, DREB2A, DREB2B, RD22, RD29A*, and *RD29B* and pointedly increases the formation of lateral root and shortens the length of the primary root during drought (Shi et al., 2014). A set of flavin monooxygenases known as YUCCAs has been discovered in various plants. These enzymes play a crucial role in tryptophan-dependent auxin biosynthesis by catalyzing the conversion of tryptamine to N-hydroxy tryptamine (Zhao et al., 2001). In Arabidopsis, *YUC7* can augment endogenous IAA levels and play several roles during drought stress. *yuc7-1D* overexpression studies had confirmed that upregulation of *YUC7* genes consequently upregulated drought resistance genes like *RD29A* and *COR15A* and increased auxin levels had modified the root system increasing lateral root numbers to tolerate the stress (Lee et al., 2012). In potato, *AtYUC6* overexpressed transgenic lines reduced ROS content significantly and improved phenotypic characters during drought conditions as compared to wild-type plants conferring the involvement of auxin in drought tolerance (Kim et al., 2013). In oilseed rape, *BnaYUC6a* overexpressing transgenic lines produced a high amount of auxin and consequently, drought-responsive genes including *ABA2*, *RD26*, and *RD29* expressed in high levels supporting auxin-mediated drought tolerance (Hao et al., 2022). In poplar and potato plants, the modulation of auxin levels has been achieved by regulating the expression of *YUCCA6* using both stress-inducible and constitutive promoters. This manipulation led to increased auxin levels and enhanced drought tolerance (Kim et al., 2013; Ke et al., 2015). During drought conditions in rice, there is an upregulation in the expression level of *OsPIN3t*, an auxin efflux carrier, indicating the role of polar auxin transport (PAT) in stress response. Consequently, it leads to the activation of drought-responsive genes, namely *OsAP37* and *OsDREB2A* (Zhang et al., 2012).

Multiple Gretchen Hagen 3 (*GH3*) family genes have been identified in different plants, including crops, where they significantly influence amide-linked IAA conjugate formation. GH3 enzymes add amino acid residues to free IAA molecules, forming conjugates that reduce the pool of active auxin available for signaling. Numerous studies have demonstrated that under drought stress conditions, the expression of these genes is upregulated (Yuan et al., 2013; Feng et al., 2015; Singh et al., 2015; Yao et al., 2023). For instance, in Arabidopsis, an activation-tagged wes1-D dwarf mutant exhibits a 44.6% reduction in free IAA levels, accompanied by a 621% increase in IAA-Asp conjugates under abiotic stress. The mutant exhibits dwarf phenotypic traits due to a markedly low level of free auxin. Additionally, its auxin-mediated lateral root development is notably impacted, resulting in a reduced number of lateral roots particularly under drought conditions. This observation underscores the significance of free auxin in the process of stress acclimatization. Further substantiating this, the application of a modest quantity of exogenous IAA has been demonstrated to augment the number of lateral roots (Park et al., 2007). One potential reason for the upregulation of *GH3* genes could be the elevated levels of abscisic acid (ABA) during drought conditions (Mittler and Blumwald, 2015) and exogenous ABA treatment also confirmed increased relative expression of GH3 genes (Park et al., 2007; Seo et al., 2009). The ABA signal transduction pathway interacts with auxin signaling, potentially suppressing auxin responses. Lowering auxin levels and hindering its signaling are anticipated to reduce growth rates in poplar plants (Popko et al., 2010). Under drought stress in rice, the expression of six *OsYUCCA* genes, and tryptophan biosynthesis anthranilate synthase genes were downregulated. Conversely, genes related to jasmonic acid (JA) biosynthesis were found to be upregulated in these conditions (Du et al., 2013). JA may act antagonistically to suppress the biosynthesis of IAA but this needs further investigation. Hence, when faced with drought stress, plants need an external source of auxin which can help the plant in tolerating this stress.

### 3.2 Auxin and salt stress

Increased soil salinity elevates the levels of Na^+^ and Cl^-^ within plants, consequently raising the Na^+^/K^+^, which disrupts normal ionic functions within plants (Singh et al., 2014). Many plants have evolved various strategies to address these challenges including phytohormonal signaling.

The IAOx pathway of auxin biosynthesis (**Figure 1B**) involves *P450* genes such as *CYP79B2* and *CYP79B3*, which have been found to positively contribute to salt tolerance. Elevated expression of these specific genes promotes lateral root development in response to salt stress (Julkowska et al., 2017). Auxin influx plays a crucial role in proper plant development and is associated with responses to salt stress (Mellor et al., 2016). Key transmembrane transporter proteins facilitating auxin influx are AUX1 (Auxin Transporter Protein 1) and LAX (Like Auxin Resistant). These proteins participate in various processes, such as gravitropic responses and the emergence of lateral roots (Swarup et al., 2008). LAX3 proteins have been associated with the salt stress response, playing an active role in lateral root development (Mellor et al., 2016). Moreover, overexpression of *WRKY3* in *Solanum lycopersicum* results in elevated levels of *LAX3* transcripts. Remarkably, enhanced resistance to salt stress is exhibited by WRKY3 overexpression lines (Hichri et al., 2017). In response to salt stress, the expression of the *YUCCA* genes is intricately regulated (Korver et al., 2018). For example, when *Cucumis sativus* plants are exposed to salt stress, there is a regulatory interplay among *CsYUC10a*, *CsYUC10b*, and *CsYUC11* genes. Under 100 mM salt stress, *CsYUC10b* experiences an increase in expression, whereas *CsYUC10a* and *CsYUC11* exhibit notable downregulation. This opposing regulation is reinforced by a complementary expression observed in specific tissues. Together, these observations indicate that this opposing mechanism serves to establish a buffering system for endogenous auxin production in cucumber during the stress conditions. Furthermore, studies have confirmed that the overexpression of *CsYUC11* leads to higher concentrations of free IAA and enhances the salt tolerance mechanisms in transgenic Arabidopsis plants (Yan et al., 2016). The function of auxin receptors has been extensively investigated in salt stress-related conditions (Julkowska et al., 2017; Bouzroud et al., 2018). IAA regulates gene expression by directly interacting with TIR/AFB receptors, leading to the SCF E3-ubiquitin ligase-mediated proteasomal degradation of Aux/IAA transcriptional repressor proteins (**Figure 1C**, Gray et al., 2001). TIR/AFB receptors are actively involved in plant’s response to salt stress. In Arabidopsis, a miR393-resistant variant of TIR1 (mTIR1) overexpression leads to enhanced salt tolerance. miR393, which targets TIR1 and AFB2 receptors for degradation is shown to increase in NaCl-induced salt stress. This degradation leads to the downregulation of auxin signaling and consequent repression of Auxin Response factor (ARF) genes (**Figure 1C**). However, the heightened expression of mTIR1 augments auxin signaling and bolsters plant resistance to salt stress by enhancing osmoregulation and augmenting Na^+^ exclusion mechanisms (Chen et al., 2015). ARF transcription factors are key players in the auxin signaling pathway as these interact with the promoters of auxin-responsive genes (Lavy and Estelle, 2016). ARFs have been identified as crucial elements in several responses of the plants to abiotic stress (Wang et al., 2010; Hu et al., 2015). The role of ARF proteins has been investigated in rice and sweet potato under salt and drought stress. Overexpression of sweet potato *IbMP/ARF* in Arabidopsis enhances auxin signaling under both drought and salt stress (Kang et al., 2018). Genes like *OsARF11* and *OsARF15* in rice are upregulated by several folds under salt stress conditions implicating their role in this response (Jain and Khurana, 2009). Furthermore, the transportation of auxin across cells within a plant necessitates a well-coordinated auxin transport system. Among the key protein efflux carriers facilitating polar auxin transport (PAT), the PIN family proteins play a central role. However, the physiological and biochemical alterations induced by salt stress adversely impact PAT, posing a potential threat to the effective functioning of the auxin transport network. Salt stress triggers an increase in phospholipase D activity, leading to the localization of clathrin in the plasma membrane. This, in turn, initiates clathrin-mediated endocytosis of PIN2 proteins. Consequently, auxin redistribution occurs, causing the root tip to bend away from areas with higher salt levels, known as auxin-mediated halotropism (Galvan-Ampudia et al., 2013). The PIN protein family, especially the plasma membrane-located proteins like PIN1, PIN3, and PIN7, are essential for controlling auxin transport and adapting to salt stress. Notably, under salt stress conditions, there is a significant impairment in auxin transport, aligning with the detrimental effects of salt stress on root development. During salt stress, nitric oxide (NO) production is triggered which directs *PIN1*, *PIN3*, and *PIN7* downregulation in Arabidopsis. This decrease in expression results in reduced auxin transport and subsequently impacts auxin signaling (Liu et al., 2015). Free auxin levels are also affected during salt stress. Notably, *GH3* genes are activated during salt stress (Korver et al., 2018). Collectively, these alterations lead to a decrease in the endogenous free auxin levels, ultimately resulting in diminished plant growth.

**Figure 2:**
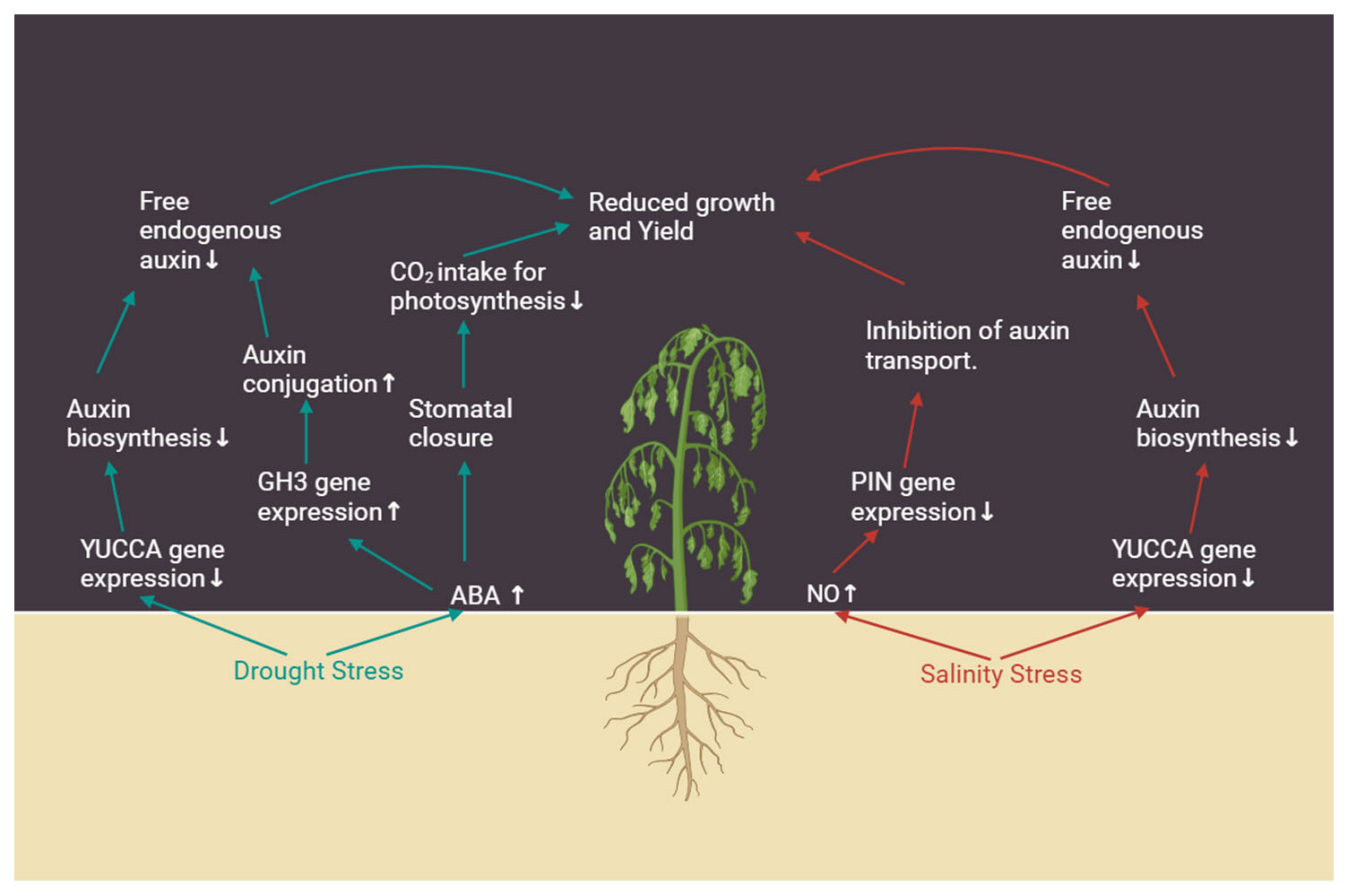
Impact of drought and salinity stress on various aspects of auxin signaling, and its consequences on gene expression and physiology. Arrows next to the text indicate increase (↑) or decrease (↓).

A brief overview of the impact of drought and salt stress on plant growth through the involvement of auxin is depicted in **Figure 2**.

## 4 Auxin-producing endophytes and their potential use in drought and salt tolerance

Several researchers have extensively investigated auxin production, particularly IAA by plant-associated microorganisms, including endophytic bacteria (Kuklinsky-Sobral et al., 2004; Madhaiyan et al., 2004; Tsavkelova et al., 2007; Khan et al., 2014). However, both drought stress and salt stress are limiting factors in the growth of such organisms thus highlighting a need to find stress-tolerant endophytes. A logical step would be to look for plants that face such stresses regularly, namely xerophytic and halophytic plants, and study their microbiome (Bokhari et al., 2019; Rodríguez-Llorente et al., 2019; ALKahtani et al., 2020; Belaouni et al., 2022; Chebotar et al., 2022). Several researchers have isolated numerous auxin-producing stress-tolerant endophytic bacteria from such plants. These bacteria were further introduced into various crop plants subjecting them to diverse stress conditions. Promising bacterial isolates with the potential to promote plant-growth parameters like auxin quantity, seed germination, and root and shoot length have been identified (Govindasamy et al., 2022; Hwang et al., 2022).

### 4.1 Drought stress mitigation using auxin-producing endophytic bacteria

Drought stress causes a decrease in auxin concentration in plants, necessitating an increased supply to alleviate the stress and sustain growth. Several research groups have investigated auxin-producing drought-tolerant endophytic bacteria, and upcoming discussions will delve into recent research findings in detail (**Table 1**). *Opuntia ficus-indica*, a desert plant, has been identified as a valuable source of multiple drought-tolerant auxin-producing endophytic bacteria, having several plant growth-promoting characteristics. Among the several *Streptomyces* species isolated, *S. rameus* VL-70-PIII demonstrated the highest auxin production, reaching a peak of 200.82 µg/ml in a medium supplemented with 100 mg/ml L-tryptophan after a 5-day period. Upon inoculating these strains to wheat seeds and keeping in drought conditions, *S. turius* VL-70-IX treatment led to a maximum increase in rootlet count. Furthermore, the co-inoculation of *S. levis* VL-70-XII and *S. turius* VL-70-IX resulted in the maximum increase in root length, while *S. mutabilis* HV-VIII showed the highest increase in shoot length (Govindasamy et al., 2022). In a separate study, an endophytic bacterium, *Pantoea alhagi*, isolated from Camelthorn plant *Alhagi sparsifolia*, exhibited drought-tolerant traits, thriving in media supplemented with 20% PEG and producing up to 17.73 µg/mL of IAA. When introduced to drought-stressed wheat seedlings, this strain effectively enhanced various plant growth parameters, including fresh weight, chlorophyll content, and soluble sugar content significantly (Chen et al., 2017). In a different study, three drought-tolerant actinobacteria from the roots of five distinct plant species (**Table 1**) significantly boosted growth and yield in another wheat cultivar, WR-544, by several folds. Instead of single isolate inoculation, co-inoculation with *Streptomyces olivaceus* and *S. geysiriensis* demonstrated maximum enhancement in growth and yield properties in drought-stressed wheat fields (Yandigeri et al., 2012).

**Table 1:** Auxin-producing endophytic bacteria, along with their sources and applications in promoting plant growth and alleviating drought stress.

Sr. No.	Endophytic Bacteria	Source plant	Plant part used for isolation of endophytes	Inoculated plant	Plant response	Reference
1	*Streptomyces turius* VL-70-IX	*Opuntia ficus-indica*	Roots	*Triticum aestivum* (cultivar- nethravati)	Increase in rootlet numbers, root length, shoot length and total seedling length	(Govindasamy et al., 2022)
2	*S. levis VL-70-XII*					
3	*S. mutabilis* HV-18					
4	*S. mutabilis* HV-VIII					
5	*S. rameus* VL-70-PIII					
6	*Micrococcus luteus* strain 4.43	*Helianthus tuberosus*	Leaf and stem	*Helianthus tuberosus*	Improved plant height, total fresh weight and dry weight, root length and diameter, and harvest index	(Namwongsa et al., 2019)
7	*Bacillus aquimaris* 3.13					
8	*B.* sp. 5.2					
9	*B. methylotrophicus* 5.18					
10	*Staphylococcus* sp. Ceb1	*Curcuma longa*	Rhizome	*Vigna unguiculata*	Increased root length and number, and shoot length	(Jayakumar et al., 2020a)
11	*Shewanella putrefaciens* strain MCL-1	*Pennisetum glaucum, Brassica nigra, Cyamopsis tetragonoloba*		*Pennisetum glaucum* (variety Pusa Composite-443)	Improved seed germination percentage, plumule length, radicle length, and fresh weight. Upregulation of drought-responsive SbNAC1, PgAP2 and PgDREB2A genes	(Manjunatha et al., 2017, Manjunatha et al., 2022)
12	*Cronobacter dublinensis* strain MKS-1					
13	*Bacillus* sp. Acb9	*Ananas comosus*	Leaf	*Vigna radiata*	Increased shoot length, root length, and root numbers	(Jayakumar et al., 2020b)
14	*Providencia* sp. Acb11					
15	*Staphylococcus* sp. *Acb12*					
16	*Staphylococcus* sp. *Acb13*					
17	*Staphylococcus* sp. *Acb14*					
18	*Acinetobacter pittii*	*Sorghum bicolor*	Root	*Sorghum bicolor* (variety CO 30 and K 30)	Increase in seed germination percentage	(Umapathi et al., 2022)
19	*Pseudacidovorax intermedius*					
20	*Exiguobacterium* sp. Sch36	*Sporobolus speccatus*, *Cyperus laevigatus*	Root, Stem and Leaves	–		(Enquahone et al., 2022)
21	*Exiguobacterium* sp. Rch312					
22	*Alishewanella* sp. Rch14					
23	*Pantoea alhagi*	*Alhagi sparsifolia*	Whole plant	*Triticum aestivum* (cultivar Yumai 49-198)	Improvement in root and shoot length, plant fresh weight, and chlorophyll, MDA, and soluble sugar content in leaves	(Chen et al., 2017)
24	*Streptomyces coelicolor* DE07	*Aerva tomentosa*, *Acacia nilotica*, *Leptadenia pyrotechnica*, *Calligonum polygonides*, *Pennisetum glaucum*	Roots	*Triticum aestivum* (cultivar WR-544)	Increased root and shoot length, tiller numbers, fresh and dry weight of root and shoot, and yield	(Yandigeri et al., 2012)
25	*S. olivaceus DE10*					
26	*S. geysiriensis DE27*					

The microbiome of medicinal plants has also been explored to identify drought-tolerant endophytic bacteria. For example, the rhizome of *Curcuma longa* harbored *Staphylococcus* sp. Ceb1, an endophyte capable of producing auxin along with other characteristics promoting plant growth. Surface-sterilized *Vigna unguiculata* seeds were germinated, treated with Ceb1, and then subjected to drought stress by withholding water for three weeks, after which water was resumed for one day before determining plant growth parameters. Compared to the control, there was an increase of 87.5% in root number, 208.4% in root length, and 55.54% in shoot length (Jayakumar et al., 2020a).

Researchers have explored the method of isolating stress-tolerant endophytes from crops and reintroducing them back into the same crops to enhance the uptake of these endophytes within the plant body, resulting in improved outcomes. These studies highlight the potential of native endophytes in stress tolerance. Manjunatha et al. (2017), isolated *Shewanella putrefaciens* MCL-1 and *Cronobacter dubliensis* MKS-1 from mustard, cluster bean, and pearl millet. Both MCL-1 and MKS-1 demonstrated the ability to promote growth. Following soaking in endophyte broth cultures for one hour, the sterilized seeds were placed on agar petri plates supplemented with 20% PEG for germination. Three days later, treatment with MCL-1 resulted in a 16.6% increase in plumule length, 9.02% increase in radicle length, and a 16.88% increase in fresh weight, while MKS-1 treatment led to an 18.8% increase in plumule length, 24% increase in radicle length, and a 21.63% increase in fresh weight (Manjunatha et al., 2017). Further investigations conducted by the same research group affirmed that under severe drought stress conditions, endophyte-inoculated pearl millet plants treated with MCL-1 and MKS-1 demonstrated the ability to elevate auxin levels in pearl millet, resulting in a significant 68%–78% increase in IAA content, compared to uninoculated controls. Moreover, they noted a substantial upregulation, by several folds, of various stress-responsive genes such as *SbSNAC1*, *PgDREB2A*, and *PgAP2* under severe drought conditions in comparison to the endophyte-uninoculated control (Manjunatha et al., 2022). Eventually, the upregulation of these defense genes is the major target for auxin-mediated defense response against abiotic stresses as discussed in earlier sections. In another study, *Micrococcus luteus* 4.43, *Bacillus aquimaris* 3.13, *Bacillus* sp. 5.2, and *B. methylotrophicus* 5.18, isolated from *Helianthus tuberosus*, exhibited the ability to produce auxin while promoting the growth of *H. tuberosus* from planting to harvesting stages under water-stressed conditions. Among these strains, *M. luteus* produced the highest amount of IAA. Strains 4.43 and 3.13 notably enhanced fresh shoot weight and plant height, respectively, when plants received only 1/3 of their water requirement at 140 days. Strain 3.13 also increased shoot and root dry weight significantly under conditions of reduced water, both at 140 days (using only 2/3 of water) and at 60 days (using only 1/3 of water). Additionally, Strain 4.43 exhibited the greatest improvement in yield under conditions of limited water supply, specifically when only 1/3 of the water requirement was provided (Namwongsa et al., 2019). In a separate study, Umapathi et al. (2022), discovered that endophytic bacteria associated with sorghum roots have the ability to produce IAA and GA, while also enhancing various plant growth parameters under drought stress conditions. Particularly, *Pseudacidovorax intermedius* demonstrated the highest production of under -1 MPa PEG 6000 stress (Umapathi et al., 2022).

Economically significant plants like *Ananas comosus* have been utilized for isolating potent endophytic bacteria. Among the five isolated strains, *Providencia* sp. Acb11 exhibited the highest auxin production of 100 µg/ml under PEG (-1.5 MPa) conditions. Whereas, *Bacillus* sp. Acb9 produced 55 µg/mL IAA and *Staphylococcus* sp. Acb13 produced 10 µg/mL IAA under the same conditions. All strains contributed to the promotion of plant growth in *Vigna radiata* seedlings*. Bacillus* sp. Acb9 notably increased shoot length and root length by 34.8% and 153%, respectively. Additionally, *Staphylococcus* sp. Acb13 significantly enhanced the maximum root number by 160% compared to the control (Jayakumar et al., 2020b).

### 4.2 Salt stress mitigation using auxin-producing endophytic bacteria

Numerous studies suggest the involvement of endophytes that produce auxin in the tolerance to salt stress conditions (**Table 2**). Many such endophytes were isolated from halophytes. In one such study conducted by Hwang et al. (2022), *Priestia megaterium* Strain BP-R2 was isolated from the halophytic plant *Bolboschoenus planiculmis*. The bacterium was capable of producing approximately 25 µg/mL of IAA in NaCl concentrations ranging from 0.5 to 3.0% over a period of 48 hours. When inoculated in *Arabidopsis thaliana* (ecotype Columbia) plants under 250 mM NaCl conditions, it led to more than a 1.5-fold increase in leaf numbers, rosette diameter, fresh weight, and dry weight compared to control plants. Similarly, inoculation of the bacteria in *Brassica rapa* (pak choi) plants under 200 mM NaCl conditions resulted in a significant increase in plant height, width, leaf numbers, total leaf area, leaf length, width, and area per leaf, as well as root fresh weight, dry weight, and length as compared to control plants (Hwang et al., 2022). In another study, *Bacillus cereus* KP120, isolated from the halophytic plant *Kosteletzkya pentacarpos* produced significant amount of IAA after 15 minutes in LB medium supplemented with Tryptophan. When inoculated in Arabidopsis seedlings under 200 mM NaCl concentration, KP120 increased the IAA concentration by 35.83% in roots and 8.41% in leaves compared to control plants. Additionally, plant height, branch number, leaf number and root lengths increased by 182.24%, 53.84%, 14.28%, and 14.40% respectively as compared to the control group (Zhang et al., 2022). In another study, Khan et al. (2020), selected six bacterial endophytes from the root tissues of *Oenothera biennis* L., *Chenopodium ficifolium* Smith, *Artemisia princeps* Pamp, *Echinochloa crus-galli* (L.). Among these, *Enterobactor ludwigii* and *Curtobacterium luteum* produced 2.7 µg/mL IAA, whereas *Enterobacter tabaci, Bacillus cereus, Micrococcus yunnanensis*, and *Micrococcus curtobacterium oceanosedimentum* produced IAA in 1.1 to 1.6 µg/mL range. All the six strains of bacteria were tested for their effect on rice plants growing under 150 mM NaCl by inoculating the roots. *M. yunnanensis* increased shoot length by a maximum of 22.9%, *M. yunnanensis* and *C. luteum* increased root length by a maximum of 40%, *M. yunnanensis* increased fresh weight by a maximum of 25.7% and *C. oceanosedimentum* increased dry weight by a maximum of 29.1% and chlorophyll content by 52.1% in comparison to control (Khan et al., 2020). The shoot-associated endophyte, *Stenotrophomonas pavanii*, isolated from the halophyte *Seidlitzia rosmarinus* could produce a maximum of 20.5 µg/ml IAA when tryptophan was added to the media. Out of total 17 endophytes, 11 endophytes were capable of producing IAA and among them 10 were capable of promoting growth in cress-lettuce. *Pseudomonas fluorescens* showed the maximum increase in seed germination percentage, root growth, and shoot growth by 9%, 16.6%, and 11.7%, respectively under 100mM NaCl stress (Shurigin et al., 2020). In a separate study, *Oceanobacillus* sp.76*, Bacillus* sp. 7, and *Micrococcus luteus* 14 were isolated from *Cressa cretica, Salsola yazdiana* and *Salsola tomentosa*, respectively. These strains demonstrated the ability to germinate seeds of *Triticum aestivum* cv. Homa and *T. aestivum* cv. Mihan up to 91.66%, while control seeds failed to germinate under 300 mM NaCl stress. Furthermore, they significantly increased seedling, root, and shoot length in both wheat varieties under NaCl treatment up to 300 mM (Soltani et al., 2024). In their study, Zhao et al. (2016), investigated the effects of endophytes, associated with the halophytic plant *Salicornia europiea* in promoting the growth of *S. europiea* under salinity stress up to 500 mM. The auxin production capability of these endophytes was also assessed, and *Planococcus rifietoensis* exhibited the highest production, reaching a maximum of 1.2 µg/mL while tolerating up to 0.68 M NaCl concentration (Zhao et al., 2016).

**Table 2:** Auxin-producing endophytic bacteria, along with their sources and applications in promoting plant growth and alleviating salt stress.

Sr. No.	Endophytic Bacteria	Source plant	Plant part used for isolation of endophytes	Inoculated plant	Plant response	Reference
1	*Priestia megaterium*	*Bolboschoenus planiculmis*	Root	*Arabidopsis thaliana*, *Brassica rapa*	Increased fresh and dry weight, leaf numbers, total leaf area and average plant height	(Hwang et al., 2022)
2	*Bacillus cereus* KP120	*Kosteletzkya pentacarpos*		*Arabidopsis thaliana*	Upregulation of several SAUR family genes, YUCCA genes, ethylene synthesis, and signaling genes. Improvement in fresh and dry weight of shoot and root, plant height, root length, branch number and leaf number	(Zhang et al., 2022)
3	*Curtobacterium oceanosedimentum*	*Oenothera biennis L*. *Artemisia princeps* Pamp. *Chenopodium ficifolium* Smith. *Echinochloa crus-galli (L.) P.Beauv.*	Root	*Oryza sativa*	Increased shoot and root length, fresh and dry weight, and leaf chlorophyll content Upregulation of OsYUCCA1 gene, and OsPIN1 gene	(Khan et al., 2020)
4	*C. luteum*					
5	*Enterobactor ludwigii*					
6	*E. tabaci*					
7	*Bacillus cereus*					
8	*Micrococcus yunnanensis*					
9	*Kochuria palustris*	*Seidlitzia rosmarinus* Ehrenb. ex Boiss	Root, Shoot	*Lepidium sativum*	Improved root and shoot length, and seed germination percentage	(Shurigin et al., 2020)
10	*Staphylococcus succinus*					
11	*Staphylococcus epidermis*					
12	*Pseudomonas baetica*					
13	*Pseudomonas fluorescens*					
14	*Paenibacillus amylolyticus*					
15	*Stenotrophomonas pavanii*					
16	*Rothia terrae*					
17	*Planomicrobium koreense*					
181	*Planomicrobium soli*					
19	*Oceanobacillus* sp. *76*	*Cressa cretica*	Root	*Triticum aestivum*	Increased seed germination percentage, seedling length, and root and shoot length	(Soltani et al., 2024)
20	*Micrococcus luteus 14*	*Salsola tomentosa*	Shoot			
21	*Bacillus* sp. *7*	*Salsola yazdiana*	Root			
22	*Bacillus tequilensis*	*Salicornia europaea*	Stem	*Salicornia europaea*	Improved seed germination, shoot and root length, and fresh weight	(Zhao et al., 2016)
23	*Planococcus rifietoensis*		Stem			
24	*Variovorax paradoxus*		Root			
25	*Streptomyces heliomycini*	*Thymus roseus*		*Gossypium hirsutum* (variety Yumian-1)	Increased shoot and root length, and root and shoot fresh weight	(Mohamad et al., 2022)
26	*Nocardiopsis dassonvillei*					
27	*Alloactinosynnema album*					
28	*Bacillus subtilis*	*Cicer arietinum*	Root	*Pisum sativum*	Improved shoot and root length, fresh and dry weight of shoot and root, total pigment content, antioxidative activity, macronutrient concentration, and ethylene concentration	(Sofy et al., 2021
29	*Pseudomonas fluorescens*					
30	*Bacillus halotolerans*	*Lilium davidii* (variety *Unicolor*)	Root	*Lilium davidi* (variety Bright Diamond)	Improvement in plant height, leaf length, leaf width, root length and root dry weight	(Gao et al., 2022)
31	*Sphingomonas paucimobilis*	*Dendrobium officinale*	Root	–	–	(Li et al., 2023)
32	*Pseudomonas pseudoalcaligenes*	*Suaeda nigra*	Root, Aerial parts	–	–	(M. Sridevi et al., 2022)
33	*Bacillus licheniformis*	*Vigna radiata*	Root, Nodules	–	–	(Bhutani et al., 2022)
34	*Enterobacter cloacae S23*	*Arachis hypogaea* (variety VRI2)	Root nodules	–	–	(Ramakrishnan et al., 2023)
35	*Streptomyces pactum*	*Limonium sinense* – – –	Root and Leaves – – –	– – – –		(Qin et al., 2014)
36	*Klebsiella pneumoniae* subsp. *rhinoscleromatis*					
37	*Serratia rubidea*					
38	*Pseudomonas brassicacearum* subsp. *brassicacearum*					
39	*Pantoea hericii*	*Limonium vulgare*	Root	*Vitis vinifera*	Increased in number of leaves, shoot length, dry weight of shoot and root	(Navarro-Torre et al., 2023)
40	*Pantoea anthophilla*	*Limonium daveaui*				
41	*Pantoea agglomerans*					
42	*Exiguobacterium* sp. Sch36	*Sporobolus speccatus*, *Cyperus laevigatus*	Root, Stem and Leaves	–	–	(Enquahone et al., 2022)
43	*Exiguobacterium* sp. Rch312					
44	*Alishewanella* sp. Rch14					

Li et al. (2023), found that the root-associated endophyte *Sphingomonas paucimobilis*, isolated from medicinal herb, *Dendrobium officinale*, produces indole-3-acetic acid (IAA) through the indole acetamide (IAM), indole acetonitrile (IAN), and indole pyruvate (IPA) pathways. Additionally, this strain demonstrated tolerance to high levels of NaCl, up to 80 g/L (Li et al., 2023). In another study, conducted by Mohamad et al. (2022) three endophytic bacteria were screened from the medicinal plant, *Thymys roseus*, demonstrating the capability to produce auxin and tolerate up to 200 µM NaCl stress. Upon inoculation of these bacteria into cotton plants, *Streptomyces atrovirens* exhibited the greatest increase in root length and weight compared to the control, while *Alloactinosynne album* caused the maximum increase in shoot length and weight (Mohamad et al., 2022).

Numerous studies have shown the salt tolerance of various endophytic species from the *Pseudomonas* and *Bacillus* genera. In a study, *Pseudomonas fluorescens* and *Bacillus subtilis*, isolated from the roots of leguminous plant *Cicer arietinum*, could produce 5.98 and 8.11 µg/ml of IAA, respectively. *B. subtilis* exhibited greater potency, increasing shoot length, fresh weight of shoots, dry weight of shoots, fresh weight of roots, and dry weight of roots by 46.52%, 48.69%, 119.17%, 109.52%, and 141.27%, respectively, compared to the control in pea plants growing under 150 mM NaCl stress (Sofy et al., 2021). In a separate study, *Bacillus halotolerans*, an endophyte isolated from *Lilium davidii* var. unicolor, exhibited tolerance to up to 6% NaCl addition in LB media, along with confirmed auxin production ability. Upon inoculation in another *Lilium* variety, bright diamond, it led to an increase in plant height, leaf length and width, root length, and dry weight by 4%, 7.6%, 2.8%, 93.6%, and 138.7%, respectively (Gao et al., 2022). Multiple salt-tolerant and auxin-producing endophytes have been identified in *Limonium sinense*. *Streptomyces pactum*, isolated from *L. sinense* leaves, produced a maximum of 8.24 mg/L of IAA and demonstrated tolerance to 7% NaCl. It was also capable of increasing *L. sinense* seed germination by 12% under 500 mM NaCl conditions in comparison to the control (Qin et al., 2014). Utilizing microbial consortia has shown promising results for enhancing halotolerance. *Pantoea hericii*, *Pantoea anthophilla*, and *Pantoea agglomerans* were isolated from various halotolerant *Limonium* sp. and *P. anthophilla* produced a maximum of 11.78 mg/L IAA. These three isolates were inoculated as a consortium into grapevine plants under salt-stress conditions Consortium inoculated plants displayed significant increase in leaf numbers and shoot length and were able to withstand salt stress effectively. Additionally, after the stress was removed, the recovery rate of the inoculated plants was significantly higher compared to the control plants (Navarro-Torre et al., 2023).

Further, multiple studies have been carried out focusing solely on the isolation of auxin-producing salt-tolerant endophytic bacteria. A study by Sridevi et al. (2022), isolated and reported a novel endophyte, *Pseudomonas pseudoalcaligenes* from *Suaeda nigra.* This strain was capable of producing 43 µg/ml IAA after 48 hour incubation period and demonstrated tolerance to NaCl up to 8% (M. Sridevi et al., 2022). Another endophyte, *Bacillus licheniformis*, isolated from *Vigna radiata*, produced 27 µg/mL IAA after 30 minutes incubation with tryptophan and exhibited tolerance to a NaCl concentration of 15% (Bhutani et al., 2022). In another study, *Enterobacter cloacae*, isolated from the root nodules of groundnut was able to produce 0.37 µg/mL IAA under 7% NaCl stress (Ramakrishnan et al., 2023). Whether this exhibition of salt tolerance and IAA production observed in such studies proves to be useful for plants, needs further investigation.

## 5 Conclusion and future perspective

**Figure 3:**
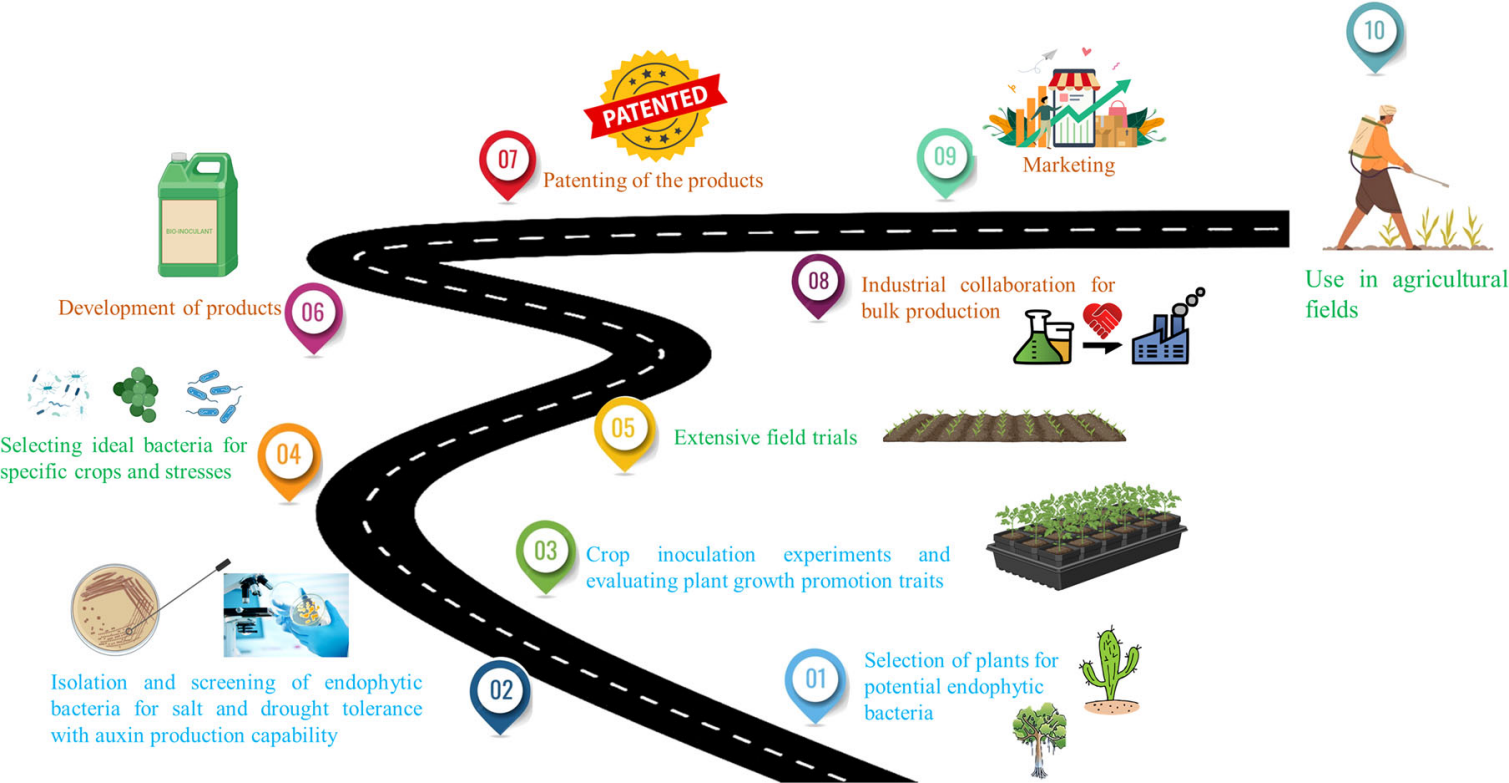
Proposed roadmap for the development of stress-specific and crop-specific bioinoculants.

Despite extensive research focusing on the isolation and screening of potential endophytes through short-term experiments, there is a notable gap in studies that span throughout the entire cultivation cycle, from sowing to harvesting of the crops to observe the effects of the potential isolates on stress alleviation and crop yield improvement. In addition, subsequent steps post-identification using the endophytes such as bioinoculant development, patenting, and marketing are imperative to make these advancements available to farmers for application in crop fields. Furthermore, it is crucial to choose an appropriate carrier for endophyte protection and stabilization during transportation and storage. Therefore, comparative studies on formulations with various carriers should be conducted to maximize the product’s effectiveness during use. To address these challenges, a suggested roadmap is delineated to guide translational research in ensuring global food security by developing bioinoculants for sustainable agricultural practices in the face of a rapidly changing climate (**Figure 3**).

A recent technological advancement in increasing agricultural productivity is the use of nanoparticles, including inorganic and organic nanomaterials. It has been reported that several endophytic bacteria produce nanomaterials, which have been demonstrated to help the plant endure abiotic stresses. Besides, using nanomaterial for bioinoculant development may enhance its effectiveness, bioavailability, and stability (Meena et al., 2021; Adeleke et al., 2022). However, the use of nanoparticles in auxin production by endophytes and auxin-mediated stress tolerance in crops needs exploration. The application of phytohormones directly using nanoparticles for plant growth promotion and defense induction has been recently explored. Recent studies have combined nanocarriers with hormones like SA, GA, JA, ABA, and IAA for the promotion of plant growth properties (Pereira et al., 2017; Clemente et al., 2018; Sun et al., 2018; Kumaraswamy et al., 2019; Korpayev et al., 2021; Gonzalez-Montfort et al., 2022; Wu et al., 2022). Future experiments that analyze the effect of nanoparticles on auxin production by endophytes and employ their use in the formulation of bioinoculants will be beneficial. This will promote studies to understand how these nanomaterials can modulate auxin biosynthesis, transport, and signaling in endophytes and plants under drought and salt stress conditions.

In conclusion, overall evidence suggests that the phytohormone auxin plays several roles in tolerating drought and salt stresses, and stress-tolerant auxin-producing endophytes can be a good source of supplemental auxin for stressed plants. This review extensively discusses and highlights potential isolates that can be used for bioinoculant development. Furthermore, the effectiveness of bioinoculants must be validated through extensive field trials in stress-affected fields before introducing the product to the market. Additionally, raising awareness among farmers to transition from conventional chemical products and using these bio-products is a crucial step towards sustainable agriculture.

## Author contributions

SM: Conceptualization, Software, Writing – original draft, Writing – review & editing. SP: Conceptualization, Funding acquisition, Project administration, Supervision, Writing – review & editing.

## References

[B1] Abdelraheem A. Esmaeili N. O’Connell M. Zhang J. (2019). Progress and perspective on drought and salt stress tolerance in cotton. Ind. Crops Prod. 130, 118–129. doi: 10.1016/j.indcrop.2018.12.070

[B2] Abobatta W. F. (2020). “Plant responses and tolerance to combined salt and drought stress,” in Salt and Drought Stress Tolerance in Plants: Signaling Networks and Adaptive Mechanisms. Eds. Hasanuzzaman M. Tanveer M. (Springer International Publishing, Cham), 17–52. doi: 10.1007/978-3-030-40277-8_2

[B3] Adeleke B. S. Akinola S. A. Adedayo A. A. Glick B. R. Babalola O. O. (2022). Synergistic relationship of endophyte-nanomaterials to alleviate abiotic stress in plants. Front. Environ. Sci. 10. doi: 10.3389/fenvs.2022.1015897

[B4] Afzal I. Shinwari Z. K. Sikandar S. Shahzad S. (2019). Plant beneficial endophytic bacteria: Mechanisms, diversity, host range and genetic determinants. Microbiol. Res. 221, 36–49. doi: 10.1016/j.micres.2019.02.001 30825940

[B5] Agarwal H. Dowarah B. Baruah P. M. Bordoloi K. S. Krishnatreya D. B. Agarwala N. (2020). Endophytes from *Gnetum gnemon* L. can protect seedlings against the infection of phytopathogenic bacterium *Ralstonia solanacearum* as well as promote plant growth in tomato. Microbiol. Res. 238, 126503. doi: 10.1016/j.micres.2020.126503 32497966

[B6] ALKahtani M. D. F. Fouda A. Attia K. A. Al-Otaibi F. Eid A. M. Ewais E. E.-D.. (2020). Isolation and characterization of plant growth promoting endophytic bacteria from desert plants and their application as bioinoculants for sustainable agriculture. Agronomy 10, 1325. doi: 10.3390/agronomy10091325

[B7] Anthelme F. Cauvy-Fraunié S. Francou B. Cáceres B. Dangles O. (2021). Living at the edge: increasing stress for plants 2–13 years after the retreat of a tropical glacier. Front. Ecol. Evol. 9. doi: 10.3389/fevo.2021.584872

[B8] Arshad M. Frankenberger W. (1991). Microbial production of plant hormones. Plant Soil 133, 1–8. doi: 10.1007/BF00011893

[B9] Bashan Y. Ream Y. Levanony H. Sade A. (1989). Nonspecific responses in plant growth, yield, and root colonization of noncereal crop plants to inoculation with *Azospirillum brasilense* Cd. Can. J. Bot. 67, 1317–1324. doi: 10.1139/b89-175

[B10] Belaouni H. A. Compant S. Antonielli L. Nikolic B. Zitouni A. Sessitsch A. (2022). In-depth genome analysis of Bacillus sp. BH32, a salt stress-tolerant endophyte obtained from a halophyte in a semiarid region. Appl. Microbiol. Biotechnol. 106, 3113–3137. doi: 10.1007/s00253-022-11907-0 35435457

[B11] Beltagy A. E. Madkour M. (2012). Impact of climate change on arid lands agriculture. Agric. Food Secur. 1, 3. doi: 10.1186/2048-7010-1-3

[B12] Bhojwani S. S. (2012). Plant Tissue Culture: Applications and Limitations (Amsterdam, Netherlands: Elsevier).

[B13] Bhutani N. Maheshwari R. Sharma N. Kumar P. Dang A. S. Suneja P. (2022). Characterization of halo-tolerant plant growth promoting endophytic Bacillus licheniformis MHN 12. J. Genet. Eng. Biotechnol. 20, 113. doi: 10.1186/s43141-022-00407-3 35920988 PMC9349330

[B14] Blakeslee J. J. Peer W. A. Murphy A. S. (2005). Auxin transport. Curr. Opin. Plant Biol. 8, 494–500. doi: 10.1016/j.pbi.2005.07.014 16054428

[B15] Bokhari A. Essack M. Lafi F. F. Andres-Barrao C. Jalal R. Alamoudi S.. (2019). Bioprospecting desert plant Bacillus endophytic strains for their potential to enhance plant stress tolerance. Sci. Rep. 9, 18154. doi: 10.1038/s41598-019-54685-y 31796881 PMC6890672

[B16] Bouzroud S. Gouiaa S. Hu N. Bernadac A. Mila I. Bendaou N.. (2018). Auxin Response Factors (ARFs) are potential mediators of auxin action in tomato response to biotic and abiotic stress (Solanum lycopersicum). PloS One 13, e0193517. doi: 10.1371/journal.pone.0193517 29489914 PMC5831009

[B17] Bulgarelli D. Schlaeppi K. Spaepen S. van Themaat E. V. L. Schulze-Lefert P. (2013). Structure and functions of the bacterial microbiota of plants. Annu. Rev. Plant Biol. 64, 807–838. doi: 10.1146/annurev-arplant-050312-120106 23373698

[B18] Chaturvedi H. Singh V. Gupta G. (2016). Potential of bacterial endophytes as plant growth promoting factors. J. Plant Pathol. 7, 7–9. doi: 10.4172/2157-7471

[B19] Chebotar V. K. Chizhevskaya E. P. Baganova M. E. Keleinikova O. V. Yuzikhin O. S. Zaplatkin A. N.. (2022). Endophytes from halotolerant plants aimed to overcome salinity and draught. Plants 11, 2992. doi: 10.3390/plants11212992 36365445 PMC9658857

[B20] Chen Z. Hu L. Han N. Hu J. Yang Y. Xiang T.. (2015). Overexpression of a miR393-resistant form of transport inhibitor response protein 1 (mTIR1) enhances salt tolerance by increased osmoregulation and Na+ exclusion in Arabidopsis thaliana. Plant Cell Physiol. 56, 73–83. doi: 10.1093/pcp/pcu149 25336111

[B21] Chen C. Xin K. Liu H. Cheng J. Shen X. Wang Y.. (2017). Pantoea alhagi, a novel endophytic bacterium with ability to improve growth and drought tolerance in wheat. Sci. Rep. 7, 1–14. doi: 10.1038/srep41564 28128318 PMC5269684

[B22] Cleland R. E. (1987). “Auxin and cell elongation,” in Plant Hormones and their Role in Plant Growth and Development. Ed. Davies P. J. (Springer Netherlands, Dordrecht), 132–148. doi: 10.1007/978-94-009-3585-3_8

[B23] Clemente I. Menicucci F. Colzi I. Sbraci L. Benelli C. Giordano C.. (2018). Unconventional and sustainable nanovectors for phytohormone delivery: insights on olea europaea. ACS Sustain. Chem. Eng. 6, 15022–15031. doi: 10.1021/acssuschemeng.8b03489

[B24] Dakora F. D. Phillips D. A. (2002). “Root exudates as mediators of mineral acquisition in low-nutrient environments,” in Food Security in Nutrient-Stressed Environments: Exploiting Plants’ Genetic Capabilities. Ed. Adu-Gyamfi J. J. (Springer Netherlands, Dordrecht), 201–213. doi: 10.1007/978-94-017-1570-6_23

[B25] Das K. Roychoudhury A. (2014). Reactive oxygen species (ROS) and response of antioxidants as ROS-scavengers during environmental stress in plants. Front. Environ. Sci. 2. doi: 10.3389/fenvs.2014.00053

[B26] Du H. Liu H. Xiong L. (2013). Endogenous auxin and jasmonic acid levels are differentially modulated by abiotic stresses in rice. Front. Plant Sci. 4. doi: 10.3389/fpls.2013.00397 PMC3793129 24130566

[B27] Du H. Wu N. Fu J. Wang S. Li X. Xiao J.. (2012). A GH3 family member, OsGH3-2, modulates auxin and abscisic acid levels and differentially affects drought and cold tolerance in rice. J. Exp. Bot. 63, 6467–6480. doi: 10.1093/jxb/ers300 23112280 PMC3504496

[B28] El-Tarabily K. A. Nassar A. H. Hardy G. E. S. J. Sivasithamparam K. (2009). Plant growth promotion and biological control of *Pythium aphanidermatum*, a pathogen of cucumber, by endophytic actinomycetes. J. Appl. Microbiol. 106, 13–26. doi: 10.1111/jam.2008.106.issue-1 19120624

[B29] Enquahone S. van Marle G. Simachew A. (2022). Plant growth-promoting characteristics of halotolerant endophytic bacteria isolated from Sporobolus specatus (Vahr) Kunth and Cyperus laevigatus L. of Ethiopian rift valley lakes. Arch. Microbiol. 204, 1–15. doi: 10.1007/s00203-022-03021-6 35723754

[B30] Esmon C. A. Pedmale U. V. Liscum E. (2005). Plant tropisms: providing the power of movement to a sessile organism. Int. J. Dev. Biol. 49, 665–674. doi: 10.1387/ijdb.052028ce 16096973

[B31] Fahad S. Bajwa A. A. Nazir U. Anjum S. A. Farooq A. Zohaib A.. (2017). Crop production under drought and heat stress: plant responses and management options. Front. Plant Sci. 8. doi: 10.3389/fpls.2017.01147 PMC5489704 28706531

[B32] FAO IFAD UNICEF WFP WHO (2022). The State of Food Security and Nutrition in the World 2022: Repurposing food and agricultural policies to make healthy diets more affordable (Rome, Italy: FAO, IFAD, UNICEF, WFP, WHO). doi: 10.4060/cc0639en

[B33] Feng S. Yue R. Tao S. Yang Y. Zhang L. Xu M.. (2015). Genome-wide identification, expression analysis of auxin-responsive *GH3* family genes in maize (*Zea mays* L.) under abiotic stresses. J. Integr. Plant Biol. 57, 783–795. doi: 10.1111/jipb.12327 25557253

[B34] Frank A. C. Saldierna Guzmán J. P. Shay J. E. (2017). Transmission of bacterial endophytes. Microorganisms 5, 70. doi: 10.3390/microorganisms5040070 29125552 PMC5748579

[B35] Galvan-Ampudia C. S. Julkowska M. M. Darwish E. Gandullo J. Korver R. A. Brunoud G.. (2013). Halotropism is a response of plant roots to avoid a saline environment. Curr. Biol. 23, 2044–2050. doi: 10.1016/j.cub.2013.08.042 24094855

[B36] Gao J. Khan M. S. Sun Y. Xue J. Du Y. Yang C.. (2022). Characterization of an endophytic antagonistic bacterial strain bacillus halotolerans LBG-1-13 with multiple plant growth-promoting traits, stress tolerance, and its effects on lily growth. BioMed. Res. Int. 2022, 5960004. doi: 10.1155/2022/5960004 36060140 PMC9436562

[B37] Gonzalez-Montfort T. S. Almaraz-Abarca N. Pérez-y-Terrón R. Ocaranza-Sánchez E. Rojas-López M. (2022). Synthesis of chitosan microparticles encapsulating bacterial cell-free supernatants and indole acetic acid, and their effects on germination and seedling growth in tomato (*Solanum lycopersicum*). Int. J. Anal. Chem. 2022, e2182783. doi: 10.1155/2022/2182783 PMC9678453 36419777

[B38] Govindasamy V. George P. Ramesh S. V. Sureshkumar P. Rane J. Minhas P. S. (2022). Characterization of root-endophytic actinobacteria from cactus (Opuntia ficus-indica) for plant growth promoting traits. Arch. Microbiol. 204, 1–14. doi: 10.1007/s00203-021-02671-2 35067746

[B39] Gray W. M. Kepinski S. Rouse D. Leyser O. Estelle M. (2001). Auxin regulates SCFTIR1-dependent degradation of AUX/IAA proteins. Nature 414, 271–276. doi: 10.1038/35104500 11713520

[B40] Gull A. Lone A. A. Wani N. U. I. (2019). “Biotic and Abiotic Stresses in Plants,” in Abiotic and Biotic Stress in Plants, (London, United Kingdom: IntechOpen). doi: 10.5772/intechopen.85832

[B41] Hallmann J. Quadt-Hallmann A. Mahaffee W. F. Kloepper J. W. (1997). Bacterial endophytes in agricultural crops. Can. J. Microbiol. 43, 895–914. doi: 10.1139/m97-131

[B42] Hao M. Wang W. Liu J. Wang H. Zhou R. Mei D.. (2022). Auxin biosynthesis genes in allotetraploid oilseed rape are essential for plant development and response to drought stress. Int. J. Mol. Sci. 23, 15600. doi: 10.3390/ijms232415600 36555242 PMC9778849

[B43] Hichri I. Muhovski Y. Žižková E. Dobrev P. I. Gharbi E. Franco-Zorrilla J. M.. (2017). The solanum lycopersicum WRKY3 transcription factor slWRKY3 is involved in salt stress tolerance in tomato. Front. Plant Sci. 8. doi: 10.3389/fpls.2017.01343 PMC5534461 28824679

[B44] Hu W. Zuo J. Hou X. Yan Y. Wei Y. Liu J.. (2015). The auxin response factor gene family in banana: genome-wide identification and expression analyses during development, ripening, and abiotic stress. Front. Plant Sci. 6. doi: 10.3389/fpls.2015.00742 PMC4569978 26442055

[B45] Hwang H.-H. Chien P.-R. Huang F.-C. Yeh P.-H. Hung S.-H. W. Deng W.-L.. (2022). A Plant Endophytic Bacterium Priestia megaterium StrainBP-R2 Isolated from the Halophyte Bolboschoenus planiculmis Enhances Plant Growth under Salt and Drought Stresses. MICROORGANISMS 10, 2047. doi: 10.3390/microorganisms10102047 36296323 PMC9610499

[B46] Iqbal M. Naveed M. Sanaullah M. Brtnicky M. Hussain M. I. Kucerik J.. (2023). Plant microbe mediated enhancement in growth and yield of canola (Brassica napus L.) plant through auxin production and increased nutrient acquisition. J. Soils Sediments 23, 1233–1249. doi: 10.1007/s11368-022-03386-7

[B47] Jahn L. Hofmann U. Ludwig-Müller J. (2021). Indole-3-Acetic Acid Is Synthesized by the Endophyte Cyanodermella asteris via a Tryptophan-Dependent and -Independent Way and Mediates the Interaction with a Non-Host Plant. Int. J. Mol. Sci. 22, 2651. doi: 10.3390/ijms22052651 33800748 PMC7961953

[B48] Jain M. Khurana J. P. (2009). Transcript profiling reveals diverse roles of auxin-responsive genes during reproductive development and abiotic stress in rice. FEBS J. 276, 3148–3162. doi: 10.1111/j.1742-4658.2009.07033.x 19490115

[B49] Jayakumar A. Krishna A. Nair I. C. Radhakrishnan E. K. (2020a). Drought-tolerant and plant growth-promoting endophytic Staphylococcus sp. having synergistic effect with silicate supplementation. Arch. Microbiol. 202, 1899–1906. doi: 10.1007/s00203-020-01911-1 32448960

[B50] Jayakumar A. Padmakumar P. Nair I. C. Radhakrishnan E. K. (2020b). Drought tolerant bacterial endophytes with potential plant probiotic effects from Ananas comosus. Biol. (Bratisl.) 75, 1769–1778. doi: 10.2478/s11756-020-00483-1

[B51] Julkowska M. M. Koevoets I. T. Mol S. Hoefsloot H. Feron R. Tester M. A.. (2017). Genetic components of root architecture remodeling in response to salt stress. Plant Cell 29, 3198–3213. doi: 10.1105/tpc.16.00680 29114015 PMC5757256

[B52] Kang C. He S. Zhai H. Li R. Zhao N. Liu Q. (2018). A sweetpotato auxin response factor gene (IbARF5) is involved in carotenoid biosynthesis and salt and drought tolerance in transgenic arabidopsis. Front. Plant Sci. 9. doi: 10.3389/fpls.2018.01307 PMC6141746 30254657

[B53] Kaur M. Karnwal A. (2023). Screening of endophytic Bacteria from stress-tolerating plants for abiotic stress tolerance and plant growth-promoting properties: Identification of potential strains for bioremediation and crop enhancement. J. Agric. Food Res. 14, 100723. doi: 10.1016/j.jafr.2023.100723

[B54] Ke Q. Wang Z. Ji C. Y. Jeong J. C. Lee H.-S. Li H.. (2015). Transgenic poplar expressing Arabidopsis YUCCA6 exhibits auxin-overproduction phenotypes and increased tolerance to abiotic stress. Plant Physiol. Biochem. 94, 19–27. doi: 10.1016/j.plaphy.2015.05.003 25980973

[B55] Keswani C. Singh S. P. Cueto L. García-Estrada C. Mezaache-Aichour S. Glare T. R.. (2020). Auxins of microbial origin and their use in agriculture. Appl. Microbiol. Biotechnol. 104, 8549–8565. doi: 10.1007/s00253-020-10890-8 32918584

[B56] Khan M. A. Asaf S. Khan A. L. Adhikari A. Jan R. Ali S.. (2020). Plant growth-promoting endophytic bacteria augment growth and salinity tolerance in rice plants. Plant Biol. 22, 850–862. doi: 10.1111/plb.13124 32329163

[B57] Khan A. L. Waqas M. Kang S.-M. Al-Harrasi A. Hussain J. Al-Rawahi A.. (2014). Bacterial endophyte Sphingomonas sp. LK11 produces gibberellins and IAA and promotes tomato plant growth. J. Microbiol. 52, 689–695. doi: 10.1007/s12275-014-4002-7 24994010

[B58] Kim J. I. Baek D. Park H. C. Chun H. J. Oh D.-H. Lee M. K.. (2013). Overexpression of arabidopsis YUCCA6 in potato results in high-auxin developmental phenotypes and enhanced resistance to water deficit. Mol. Plant 6, 337–349. doi: 10.1093/mp/sss100 22986790

[B59] Knudsen C. Janeshawari Gallage N. Cetti Hansen C. Lindberg Møller B. Laursen T. (2018). Dynamic metabolic solutions to the sessile life style of plants. Nat. Prod. Rep. 35, 1140–1155. doi: 10.1039/C8NP00037A 30324199 PMC6254060

[B60] Koepfli J. B. Thimann K. V. Went F. W. (1938). Phytohormones: structure and physiological activity. I. J. Biol. Chem. 122, 763–780. doi: 10.1016/S0021-9258(18)74205-1

[B61] Korpayev S. Karakeçili A. Dumanoğlu H. Ibrahim Ahmed Osman S. (2021). Chitosan and silver nanoparticles are attractive auxin carriers: A comparative study on the adventitious rooting of microcuttings in apple rootstocks. Biotechnol. J. 16, 2100046. doi: 10.1002/biot.202100046 34028191

[B62] Korver R. A. Koevoets I. T. Testerink C. (2018). Out of shape during stress: A key role for auxin. Trends Plant Sci. 23, 783–793. doi: 10.1016/j.tplants.2018.05.011 29914722 PMC6121082

[B63] Kramer E. M. Bennett M. J. (2006). Auxin transport: a field in flux. Trends Plant Sci. 11, 382–386. doi: 10.1016/j.tplants.2006.06.002 16839804

[B64] Kuklinsky-Sobral J. Araújo W. L. Mendes R. Geraldi I. O. Pizzirani-Kleiner A. A. Azevedo J. L. (2004). Isolation and characterization of soybean-associated bacteria and their potential for plant growth promotion. Environ. Microbiol. 6, 1244–1251. doi: 10.1111/j.1462-2920.2004.00658.x 15560822

[B65] Kumaraswamy R. V. Kumari S. Choudhary R. C. Sharma S. S. Pal A. Raliya R.. (2019). Salicylic acid functionalized chitosan nanoparticle: A sustainable biostimulant for plant. Int. J. Biol. Macromol. 123, 59–69. doi: 10.1016/j.ijbiomac.2018.10.202 30389525

[B66] Kushwaha P. Kashyap P. L. Bhardwaj A. K. Kuppusamy P. Srivastava A. K. Tiwari R. K. (2020). Bacterial endophyte mediated plant tolerance to salinity: growth responses and mechanisms of action. World J. Microbiol. Biotechnol. 36, 26. doi: 10.1007/s11274-020-2804-9 31997078

[B67] Lavy M. Estelle M. (2016). Mechanisms of auxin signaling. Development 143, 3226–3229. doi: 10.1242/dev.131870 27624827 PMC5047657

[B68] Lee M. Jung J.-H. Han D.-Y. Seo P. J. Park W. J. Park C.-M. (2012). Activation of a flavin monooxygenase gene YUCCA7 enhances drought resistance in Arabidopsis. Planta 235, 923–938. doi: 10.1007/s00425-011-1552-3 22109847

[B69] Li J. Wu H. Pu Q. Zhang C. Chen Y. Lin Z.. (2023). Complete genome of Sphingomonas paucimobilis ZJSH1, an endophytic bacterium from Dendrobium officinale with stress resistance and growth promotion potential. Arch. Microbiol. 205, 1–12. doi: 10.1007/s00203-023-03459-2 36959350

[B70] Liu W. Li R.-J. Han T.-T. Cai W. Fu Z.-W. Lu Y.-T. (2015). Salt stress reduces root meristem size by nitric oxide-mediated modulation of auxin accumulation and signaling in arabidopsis. Plant Physiol. 168, 343–356. doi: 10.1104/pp.15.00030 25818700 PMC4424022

[B71] Ludwig-Müller J. (2011). Auxin conjugates: their role for plant development and in the evolution of land plants. J. Exp. Bot. 62, 1757–1773. doi: 10.1093/jxb/erq412 21307383

[B72] Madhaiyan M. Saravanan V. S. Jovi D. Lee H. Thenmozhi R. Hari K.. (2004). Occurrence of *Gluconacetobacter diazotrophicus* in tropical and subtropical plants of Western Ghats, India. Microbiol. Res. 159, 233–243. doi: 10.1016/j.micres.2004.04.001 15462523

[B73] Manjunatha B. S. Asha A. D. Nivetha N. deppa B. Govindasamy V. Rathi M. S.. (2017). Evaluation of Endophytic Bacteria for their Influence on Plant Growth and Seed Germination under Water Stress Conditions. Int. J. Curr. Microbiol. Appl. Sci. 6, 4061–4067. doi: 10.20546/ijcmas.2017.611.475

[B74] Manjunatha B. S. Nivetha N. Krishna G. K. Elangovan A. Pushkar S. Chandrashekar N.. (2022). Plant growth-promoting rhizobacteria Shewanella putrefaciens and Cronobacter dublinensis enhance drought tolerance of pearl millet by modulating hormones and stress-responsive genes. Physiol. Plant 174, e13676. doi: 10.1111/ppl.13676 35316540

[B75] Mano Y. Nemoto K. (2012). The pathway of auxin biosynthesis in plants. J. Exp. Bot. 63, 2853–2872. doi: 10.1093/jxb/ers091 22447967

[B76] Meena M. Zehra A. Swapnil P. Harish Marwal A. Yadav G.. (2021). Endophytic nanotechnology: an approach to study scope and potential applications. Front. Chem. 9. doi: 10.3389/fchem.2021.613343 PMC8185355 34113600

[B77] Mellor N. Bennett M. J. King J. R. (2016). GH3-mediated auxin conjugation can result in either transient or oscillatory transcriptional auxin responses. Bull. Math. Biol. 78, 210–234. doi: 10.1007/s11538-015-0137-x 26767838

[B78] Mittler R. Blumwald E. (2015). The roles of ROS and ABA in systemic acquired acclimation. Plant Cell 27, 64–70. doi: 10.1105/tpc.114.133090 25604442 PMC4330577

[B79] Mohamad O. A. A. Liu Y.-H. Li L. Ma J.-B. Huang Y. Gao L.. (2022). Synergistic plant-microbe interactions between endophytic actinobacteria and their role in plant growth promotion and biological control of cotton under salt stress. Microorganisms 10, 867. doi: 10.3390/microorganisms10050867 35630312 PMC9143301

[B80] Muday G. K. (2001). Auxins and tropisms. J. Plant Growth Regul. 20, 226–243. doi: 10.1007/s003440010027 12033223

[B81] Namwongsa J. Jogloy S. Vorasoot N. Boonlue S. Riddech N. Mongkolthanaruk W. (2019). Endophytic Bacteria Improve Root Traits, Biomass and Yield of Helianthus tuberosus L. under Normal and Deficit Water Conditi. J. Microbiol. Biotechnol. 29, 1777–1789. doi: 10.4014/jmb.1903.03062 31546292

[B82] Nassar A. H. El-Tarabily K. A. Sivasithamparam K. (2005). Promotion of plant growth by an auxin-producing isolate of the yeast Williopsis saturnus endophytic in maize (Zea mays L.) roots. Biol. Fertil. Soils 42, 97–108. doi: 10.1007/s00374-005-0008-y

[B83] Navarro-Torre S. Ferrario S. Caperta A. D. Victorino G. Bailly M. Sousa V.. (2023). Halotolerant endophytes promote grapevine regrowth after salt-induced defoliation. J. Plant Interact. 18, 2215235 doi: 10.1080/17429145.2023.2215235

[B84] Nowak J. Asiedu S. K. Lazarovits G. Pillay V. Stewart A. Smith C. Liu Z. (1995). “Enhancement of in vitro growth and transplant stress tolerance of potato and vegetable plantlets co-cultured with a plant growth promoting pseudomonad bacterium,” In Proceedings of the International Symposium on Ecophysiology and Photosynthetic In Vitro Cultures., Edited by: Carre, F. and Chagvardieff, pp. 173–180. Cadarache, France: Aix-en-Provence, France.

[B85] Park J.-E. Park J.-Y. Kim Y.-S. Staswick P. E. Jeon J. Yun J.. (2007). GH3-mediated auxin homeostasis links growth regulation with stress adaptation response in arabidopsis*. J. Biol. Chem. 282, 10036–10046. doi: 10.1074/jbc.M610524200 17276977

[B86] Patel J. K. Archana G. (2017). Diverse culturable diazotrophic endophytic bacteria from Poaceae plants show cross-colonization and plant growth promotion in wheat. Plant Soil 417, 99–116. doi: 10.1007/s11104-017-3244-7

[B87] Pattison R. J. Csukasi F. Catalá C. (2014). Mechanisms regulating auxin action during fruit development. Physiol. Plant 151, 62–72. doi: 10.1111/ppl.12142 24329770

[B88] Pavlo A. Leonid O. Iryna Z. Natalia K. Maria P. A. (2011). Endophytic bacteria enhancing growth and disease resistance of potato (*Solanum tuberosum* L.). Biol. CONTROL 56, 43–49. doi: 10.1016/j.biocontrol.2010.09.014

[B89] Penuelas J. Iolanda F. Comas P. E. (2002). Changed plant and animal life cycles from 1952 to 2000 in the Mediterranean region. Glob. Change Biol. 8, 531–544. doi: 10.1046/j.1365-2486.2002.00489.x

[B90] Pereira A. E. S. Silva P. M. Oliveira J. L. Oliveira H. C. Fraceto L. F. (2017). Chitosan nanoparticles as carrier systems for the plant growth hormone gibberellic acid. Colloids Surf. B Biointerfaces 150, 141–152. doi: 10.1016/j.colsurfb.2016.11.027 27914250

[B91] Pillay V. K. Nowak J. (1997). Inoculum density, temperature, and genotype effects on in *vitro* growth promotion and epiphytic and endophytic colonization of tomato (Lycopersicon esculentum L.) seedlings inoculated with a pseudomonad bacterium. Can. J. Microbiol. 43, 354–361. doi: 10.1139/m97-049

[B92] Popko J. Hänsch R. Mendel R.-R. Polle A. Teichmann T. (2010). The role of abscisic acid and auxin in the response of poplar to abiotic stress. Plant Biol. 12, 242–258. doi: 10.1111/j.1438-8677.2009.00305.x 20398232

[B93] Porter W. L. Thimann K. V. (1965). Molecular requirements for auxin action—I.: Halogenated indoles and indoleacetic acid. Phytochemistry 4, 229–243. doi: 10.1016/S0031-9422(00)86169-5

[B94] Qin S. Zhang Y.-J. Yuan B. Xu P.-Y. Xing K. Wang J.. (2014). Isolation of ACC deaminase-producing habitat-adapted symbiotic bacteria associated with halophyte Limonium sinense (Girard) Kuntze and evaluating their plant growth-promoting activity under salt stress. Plant Soil 374, 753–766. doi: 10.1007/s11104-013-1918-3

[B95] Quadt-Hallmann A. Kloepper J. W. Benhamou N. (1997). Bacterial endophytes in cotton: mechanisms of entering the plant. Can. J. Microbiol. 43, 577–582. doi: 10.1139/m97-081

[B96] Ramakrishnan P. Ariyan M. Rangasamy A. Rajasekaran R. Ramasamy K. Murugaiyan S.. (2023). Draft genome sequence of enterobacter cloacae S23 a plant growthpromoting passenger endophytic bacterium isolated from groundnut nodule possesses stress tolerance traits. Curr. Genomics 24, 36–47. doi: 10.2174/1389202924666230403123208 37920731 PMC10334703

[B97] Riggs P. J. Chelius M. K. Iniguez A. L. Kaeppler S. M. Triplett E. W. (2001). Enhanced maize productivity by inoculation with diazotrophic bacteria. Funct. Plant Biol. 28, 829–836. doi: 10.1071/PP01045

[B98] Rodríguez-Llorente I. D. Pajuelo E. Navarro-Torre S. Mesa-Marín J. Caviedes M. A. (2019). “Bacterial endophytes from halophytes: how do they help plants to alleviate salt stress?,” in Saline Soil-based Agriculture by Halotolerant Microorganisms. Eds. Kumar M. Etesami H. Kumar V. (Springer, Singapore), 147–160. doi: 10.1007/978-981-13-8335-9_6

[B99] Rosbakh S. Leingärtner A. Hoiss B. Krauss J. Steffan-Dewenter I. Poschlod P. (2017). Contrasting effects of extreme drought and snowmelt patterns on mountain plants along an elevation gradient. Front. Plant Sci. 8. doi: 10.3389/fpls.2017.01478 PMC5581835 28900434

[B100] Sabagh A. E. Islam M. S. Hossain A. Iqbal M. A. Mubeen M. Waleed M.. (2022). Phytohormones as growth regulators during abiotic stress tolerance in plants. Front. Agron. 4. doi: 10.3389/fagro.2022.765068

[B101] Santoyo G. Moreno-Hagelsieb G. Orozco-Mosqueda Mdel C. Glick B. R. (2016). Plant growth-promoting bacterial endophytes. Microbiol. Res. 183, 92–99. doi: 10.1016/j.micres.2015.11.008 26805622

[B102] Seo P. J. Xiang F. Qiao M. Park J.-Y. Lee Y. N. Kim S.-G.. (2009). The MYB96 transcription factor mediates abscisic acid signaling during drought stress response in arabidopsis. Plant Physiol. 151, 275–289. doi: 10.1104/pp.109.144220 19625633 PMC2735973

[B103] Shaalan R. S. Gerges E. Habib W. Ibrahim L. (2021). Endophytic colonization by *Beauveria bassiana* and *Metarhizium anisopliae* induces growth promotion effect and increases the resistance of cucumber plants against *Aphis gossypii* . J. Plant Prot. Res. 61, 358–370. doi: 10.24425/jppr.2021.139244

[B104] Shi H. Chen L. Ye T. Liu X. Ding K. Chan Z. (2014). Modulation of auxin content in Arabidopsis confers improved drought stress resistance. Plant Physiol. Biochem. 82, 209–217. doi: 10.1016/j.plaphy.2014.06.008 24992887

[B105] Shi Y. Lou K. Li C. (2009). Promotion of plant growth by phytohormone-producing endophytic microbes of sugar beet. Biol. Fertil. Soils 45, 645–653. doi: 10.1007/s00374-009-0376-9

[B106] Shurigin V. Egamberdieva D. Li L. Davranov K. Panosyan H. Birkeland N.-K.. (2020). Endophytic bacteria associated with halophyte Seidlitzia rosmarinus Ehrenb. ex Boiss. from saline soil of Uzbekistan and their plant beneficial traits. J. ARID LAND 12, 730–740. doi: 10.1007/s40333-020-0019-4

[B107] Siddique S. Naveed M. Yaseen M. Shahbaz M. (2022). Exploring potential of seed endophytic bacteria for enhancing drought stress resilience in maize (Zea mays L.). Sustainability 14, 673. doi: 10.3390/su14020673

[B108] Singh V. K. Jain M. Garg R. (2015). Genome-wide analysis and expression profiling suggest diverse roles of GH3 genes during development and abiotic stress responses in legumes. Front. Plant Sci. 5. doi: 10.3389/fpls.2014.00789 PMC4294127 25642236

[B109] Singh M. Kumar J. Singh V. P. Prasad S. M. (2014). Plant tolerance mechanism against salt stress: the nutrient management approach. Biochem. Pharmacol. Open Access 03, e165. doi: 10.4172/2167-0501.1000e165

[B110] Sofy M. R. Aboseidah A. A. Heneidak S. A. Ahmed H. R. (2021). ACC deaminase containing endophytic bacteria ameliorate salt stress in Pisum sativum through reduced oxidative damage and induction of antioxidative defense systems. Environ. Sci. pollut. Res. 28, 40971–40991. doi: 10.1007/s11356-021-13585-3 33772716

[B111] Soltani J. Samavati R. Jalili B. Bagheri H. Hamzei J. (2024). Halotolerant endophytic bacteria from desert-adapted halophyte plants alleviate salinity stress in germinating seeds of the common wheat Triticum aestivum L. Cereal Res. Commun. 52, 165–175. doi: 10.1007/s42976-023-00377-3

[B112] Sridevi M. Sandhya Deepika D. Lavanya J. (2022). Isolation and Categorization of Plant Growth Promoting Endophytic Bacteria Isolated from Halophytic Suaeda nigra at Salt Stress Area of Srikakulam, Andhra Pradesh. J. Pure Appl. Microbiol. 2826, 2835–175. doi: 10.22207/JPAM

[B113] Sukumar P. Legué V. Vayssières A. Martin F. Tuskan G. A. Kalluri U. C. (2013). Involvement of auxin pathways in modulating root architecture during beneficial plant–microorganism interactions. Plant Cell Environ. 36, 909–919. doi: 10.1111/pce.12036 23145472

[B114] Sun D. Hussain H. I. Yi Z. Rookes J. E. Kong L. Cahill D. M. (2018). Delivery of abscisic acid to plants using glutathione responsive mesoporous silica nanoparticles. J. Nanosci. Nanotechnol. 18, 1615–1625. doi: 10.1166/jnn.2018.14262 29448638

[B115] Sun F. Zhang W. Hu H. Li B. Wang Y. Zhao Y.. (2008). Salt modulates gravity signaling pathway to regulate growth direction of primary roots in arabidopsis. Plant Physiol. 146, 178–188. doi: 10.1104/pp.107.109413 18024552 PMC2230569

[B116] Swarup K. Benková E. Swarup R. Casimiro I. Péret B. Yang Y.. (2008). The auxin influx carrier LAX3 promotes lateral root emergence. Nat. Cell Biol. 10, 946–954. doi: 10.1038/ncb1754 18622388

[B117] Synek L. Rawat A. L’Haridon F. Weisskopf L. Saad M. M. Hirt H. (2021). Multiple strategies of plant colonization by beneficial endophytic Enterobacter sp. SA187. Environ. Microbiol. 23, 6223–6240. doi: 10.1111/1462-2920.15747 34472197

[B118] Tsavkelova E. A. Cherdyntseva T. A. Botina S. G. Netrusov A. I. (2007). Bacteria associated with orchid roots and microbial production of auxin. Microbiol. Res. 162, 69–76. doi: 10.1016/j.micres.2006.07.014 17140781

[B119] Umapathi M. Chandrasekhar C. N. Senthil A. Kalaiselvi T. Santhi R. Ravikesavan R. (2022). Isolation, characterization and plant growth-promoting effects of sorghum [Sorghum bicolor (L.) moench] root-associated rhizobacteria and their potential role in drought mitigation. Arch. Microbiol. 204, 1–14. doi: 10.1007/s00203-022-02939-1 35641831

[B120] Vecerova K. Oravec M. Puranik S. Findurova H. Vesela B. Opoku E.. (2022). Single and interactive effects of variables associated with climate change on wheat metabolome. Front. Plant Sci. 13. doi: 10.3389/fpls.2022.1002561 PMC9589161 36299781

[B121] Verma P. Hiremani N. S. Gawande S. P. Sain S. K. Nagrale D. T. Narkhedkar N. G.. (2022). Modulation of plant growth and antioxidative defense system through endophyte biopriming in cotton (Gossypium spp.) and non-host crops. HELIYON 8, e09487. doi: 10.1016/j.heliyon.2022.e09487 35663737 PMC9157003

[B122] Walitang D. I. Kim K. Madhaiyan M. Kim Y. K. Kang Y. Sa T. (2017). Characterizing endophytic competence and plant growth promotion of bacterial endophytes inhabiting the seed endosphere of Rice. BMC Microbiol. 17, 1–13. doi: 10.1186/s12866-017-1117-0 29073903 PMC5658939

[B123] Wang S. Bai Y. Shen C. Wu Y. Zhang S. Jiang D.. (2010). Auxin-related gene families in abiotic stress response in Sorghum bicolor. Funct. Integr. Genomics 10, 533–546. doi: 10.1007/s10142-010-0174-3 20499123

[B124] Went F. W. Thimann K. V. (1937). Phytohormones. Available at: https://www.cabdirect.org/cabdirect/abstract/19381601765 (Accessed December 7, 2023).

[B125] Wu X. Hu Q. Liang X. Chen J. Huan C. Fang S. (2022). Methyl jasmonate encapsulated in protein-based nanoparticles to enhance water dispersibility and used as coatings to improve cherry tomato storage. Food Packag. Shelf Life 33, 100925. doi: 10.1016/j.fpsl.2022.100925

[B126] Yan S. Che G. Ding L. Chen Z. Liu X. Wang H.. (2016). Different cucumber CsYUC genes regulate response to abiotic stresses and flower development. Sci. Rep. 6, 20760. doi: 10.1038/srep20760 26857463 PMC4746583

[B127] Yandigeri M. S. Meena K. K. Singh D. Malviya N. Singh D. P. Solanki M. K.. (2012). Drought-tolerant endophytic actinobacteria promote growth of wheat (*Triticum aestivum*) under water stress conditions. Plant Growth Regul. 68, 411–420. doi: 10.1007/s10725-012-9730-2

[B128] Yao P. Zhang C. Qin T. Liu Y. Liu Z. Xie X.. (2023). Comprehensive analysis of GH3 gene family in potato and functional characterization of stGH3.3 under drought stress. Int. J. Mol. Sci. 24, 15122. doi: 10.3390/ijms242015122 37894803 PMC10606756

[B129] Yu J. Zhang Y. Liu W. Wang H. Wen S. Zhang Y.. (2020). Molecular evolution of auxin-mediated root initiation in plants. Mol. Biol. Evol. 37, 1387–1393. doi: 10.1093/molbev/msz202 31504735

[B130] Yuan H. Zhao K. Lei H. Shen X. Liu Y. Liao X.. (2013). Genome-wide analysis of the GH3 family in apple (Malus × domestica). BMC Genomics 14, 297. doi: 10.1186/1471-2164-14-297 23638690 PMC3653799

[B131] Zhang Q. Li J. Zhang W. Yan S. Wang R. Zhao J.. (2012). The putative auxin efflux carrier OsPIN3t is involved in the drought stress response and drought tolerance. Plant J. 72, 805–816. doi: 10.1111/j.1365-313X.2012.05121.x 22882529

[B132] Zhang X. S. O’Neill S. D. (1993). Ovary and gametophyte development are coordinately regulated by auxin and ethylene following pollination. Plant Cell 5, 403–418. doi: 10.1105/tpc.5.4.403 12271070 PMC160280

[B133] Zhang Y. Tian Z. Xi Y. Wang X. Chen S. He M.. (2022). Improvement of salt tolerance of Arabidopsis thaliana seedlings inoculated with endophytic Bacillus cereus KP120. J. Plant Interact. 17, 884–893. doi: 10.1080/17429145.2022.2111471

[B134] Zhao Y. Christensen S. K. Fankhauser C. Cashman J. R. Cohen J. D. Weigel D.. (2001). A role for flavin monooxygenase-like enzymes in auxin biosynthesis. Science 291, 306–309. doi: 10.1126/science.291.5502.306 11209081

[B135] Zhao S. Zhou N. Zhao Z.-Y. Zhang K. Wu G.-H. Tian C.-Y. (2016). Isolation of Endophytic Plant Growth-Promoting Bacteria Associated with the Halophyte Salicornia europaea and Evaluation of their Promoting Activity Under Salt Stress. Curr. Microbiol. 73, 574–581. doi: 10.1007/s00284-016-1096-7 27447799

